# Supplementary figures and images for: Modulation of bacterial multicellularity via spatio-specific polysaccharide secretion
Source: PLoS Biol. 2020 Jun 9;18(6):e3000728. doi: 10.1371/journal.pbio.3000728 (PMC7310880; doi:10.1371/journal.pbio.3000728)

# EPS cluster

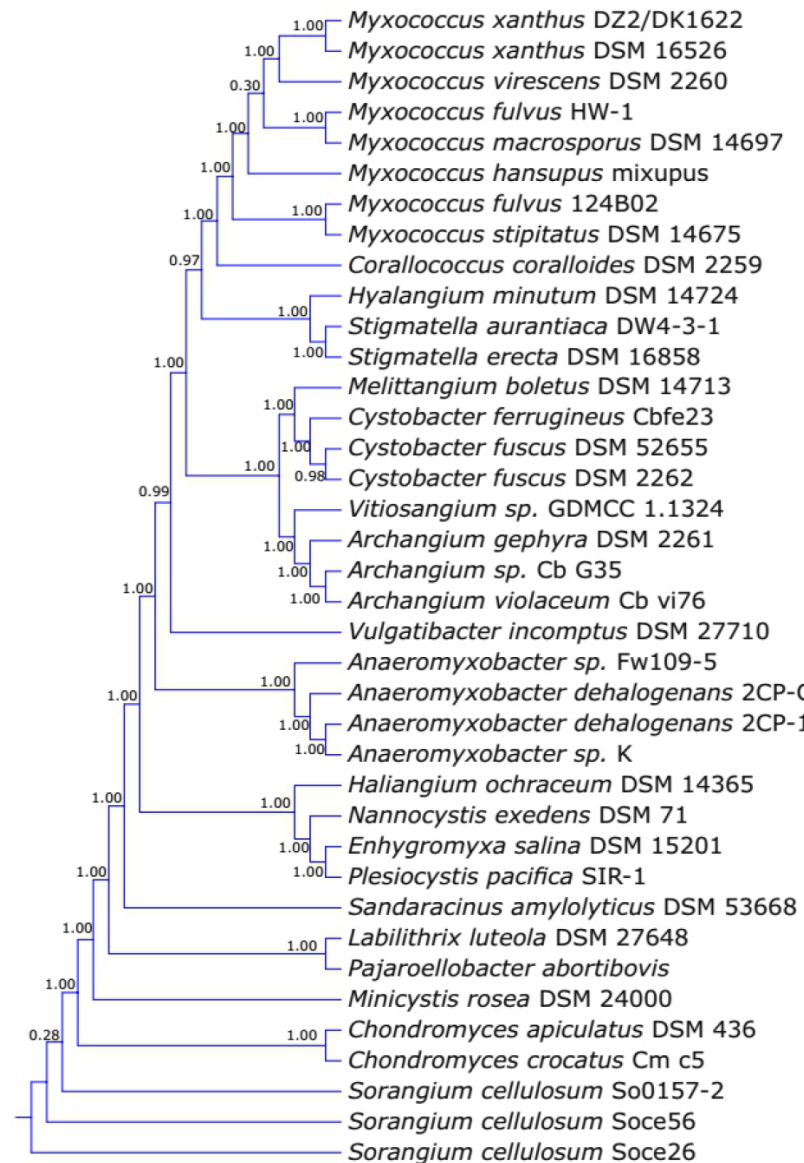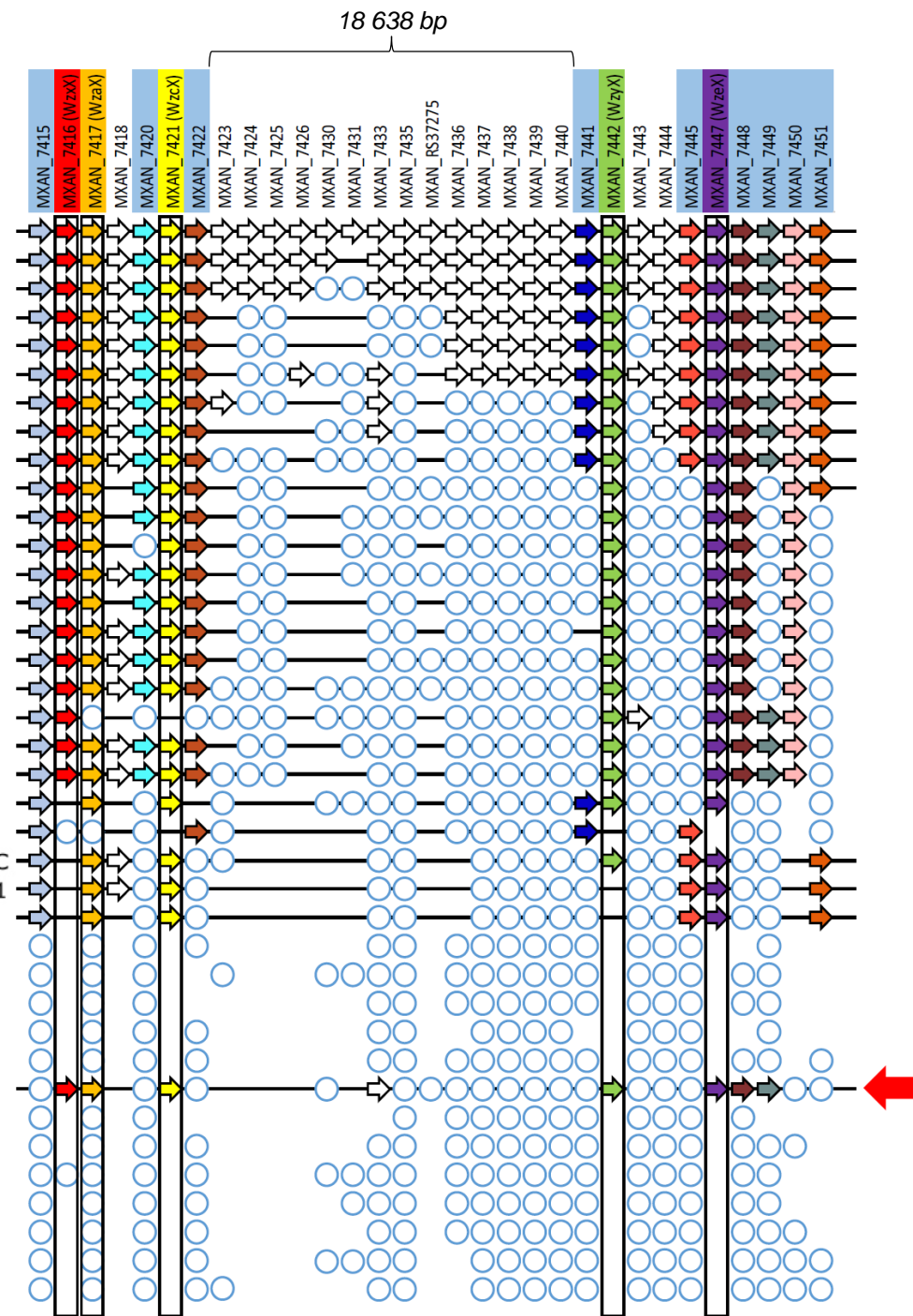

MASC cluster

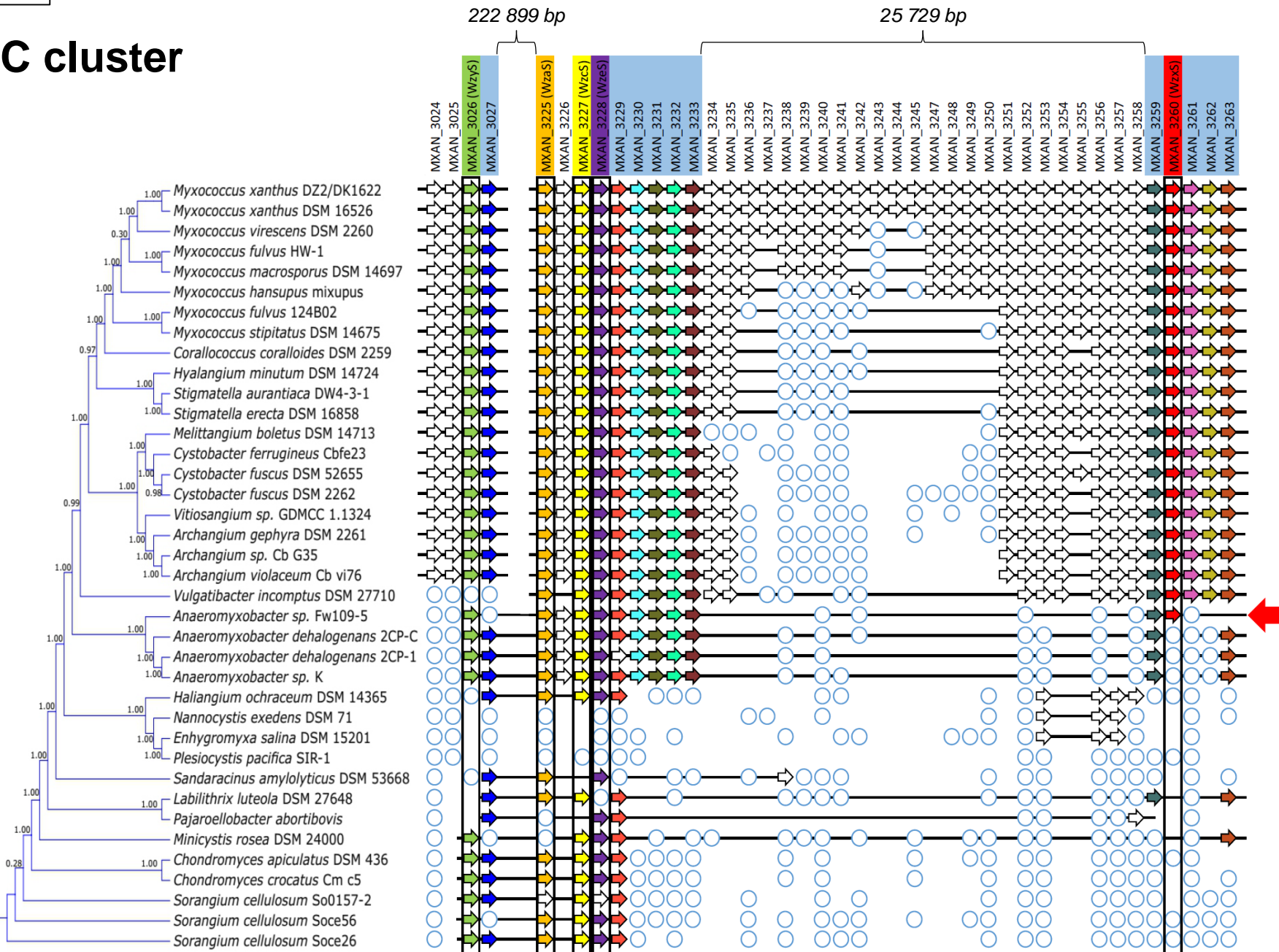

# BPS cluster

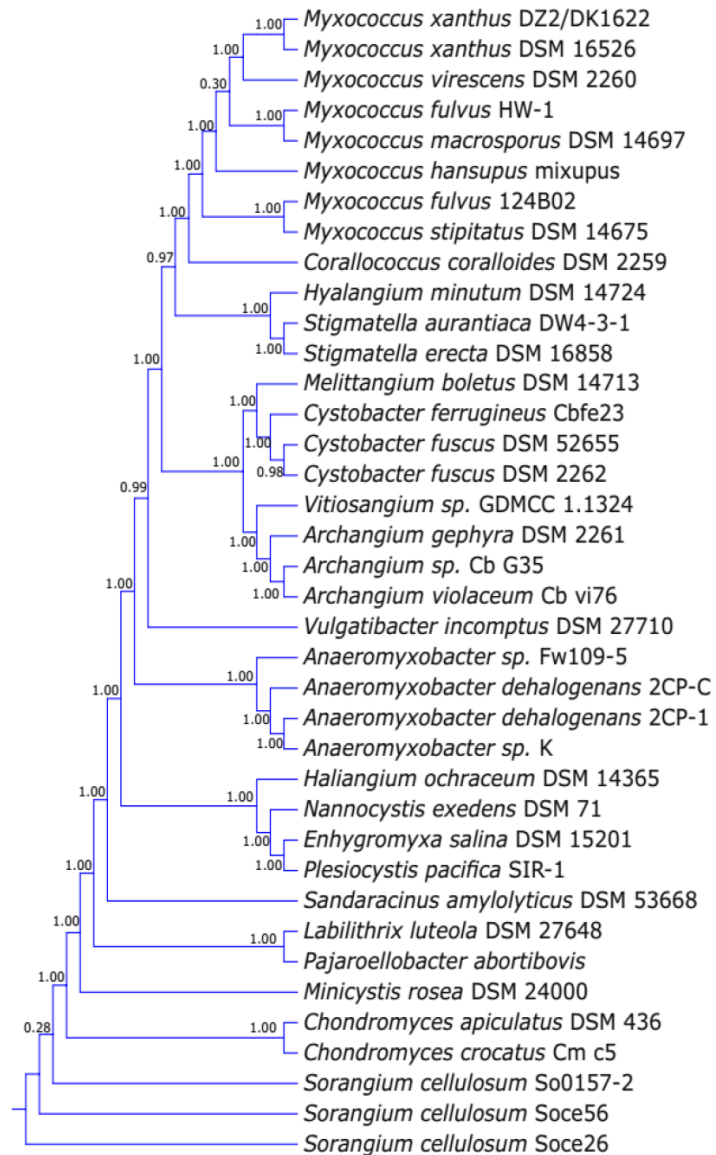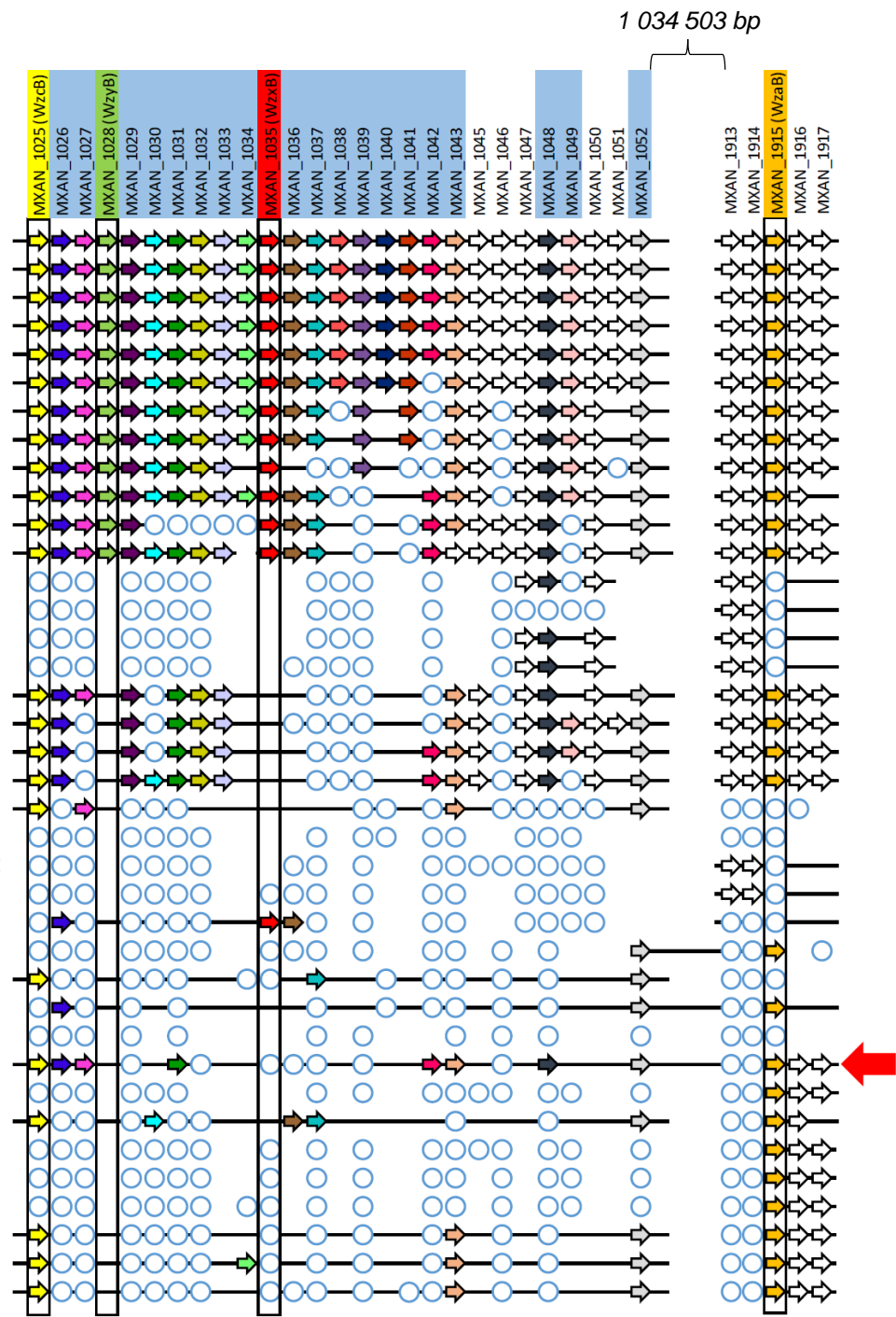

Supplement: S1 Fig — (A) EPS-, (B) MASC-, and (C) BPS-cluster evolution: core assembly-and-export constituents of each biosynthesis pathway were identified using Pfam domain scanning and BLAST-based homology searches. Based on the genomic locations of core and other neighborhood genes in M. xanthus, clusters were generated, and their binary distribution was further mapped on to the phylogenetic tree generated via aligning and concatenating 30 housekeeping proteins as shown in this figure. Arrows represent the uninterrupted presence of genes in a cluster. Locus tags highlighted by pale blue boxes correspond to genes such as enzymes involved in monosaccharide synthesis, modification, or incorporation into precursor repeat units of the respective polymer. White circles depict the presence of a homologous gene encoded elsewhere in the chromosome (but not syntenic with the remainder of the EPS/MASC/BPS biosynthesis cluster). Bootstrap values are provided on the tree nodes. BLAST, Basic Local Alignment Search Tool; BPS, biosurfactant polysaccharide; EPS, exopolysaccharide; MASC, major spore coat polysaccharide. (PDF) [file pbio.3000728.s001.pdf]

**A**

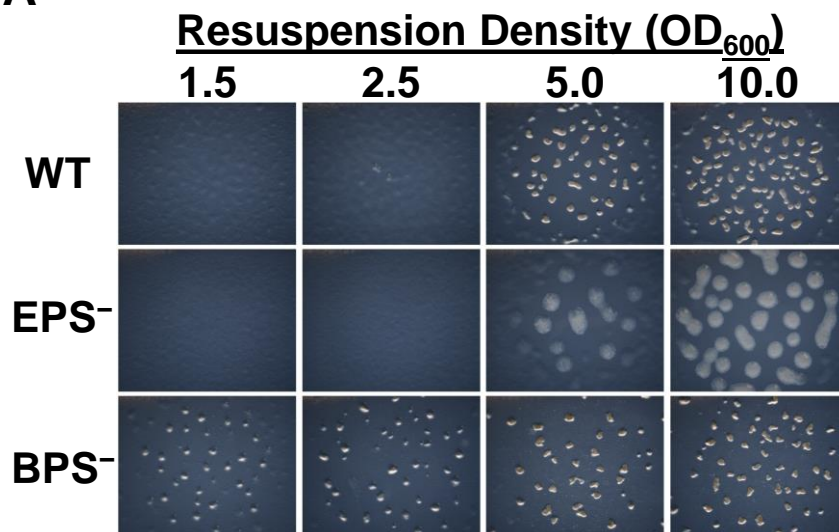

**B**

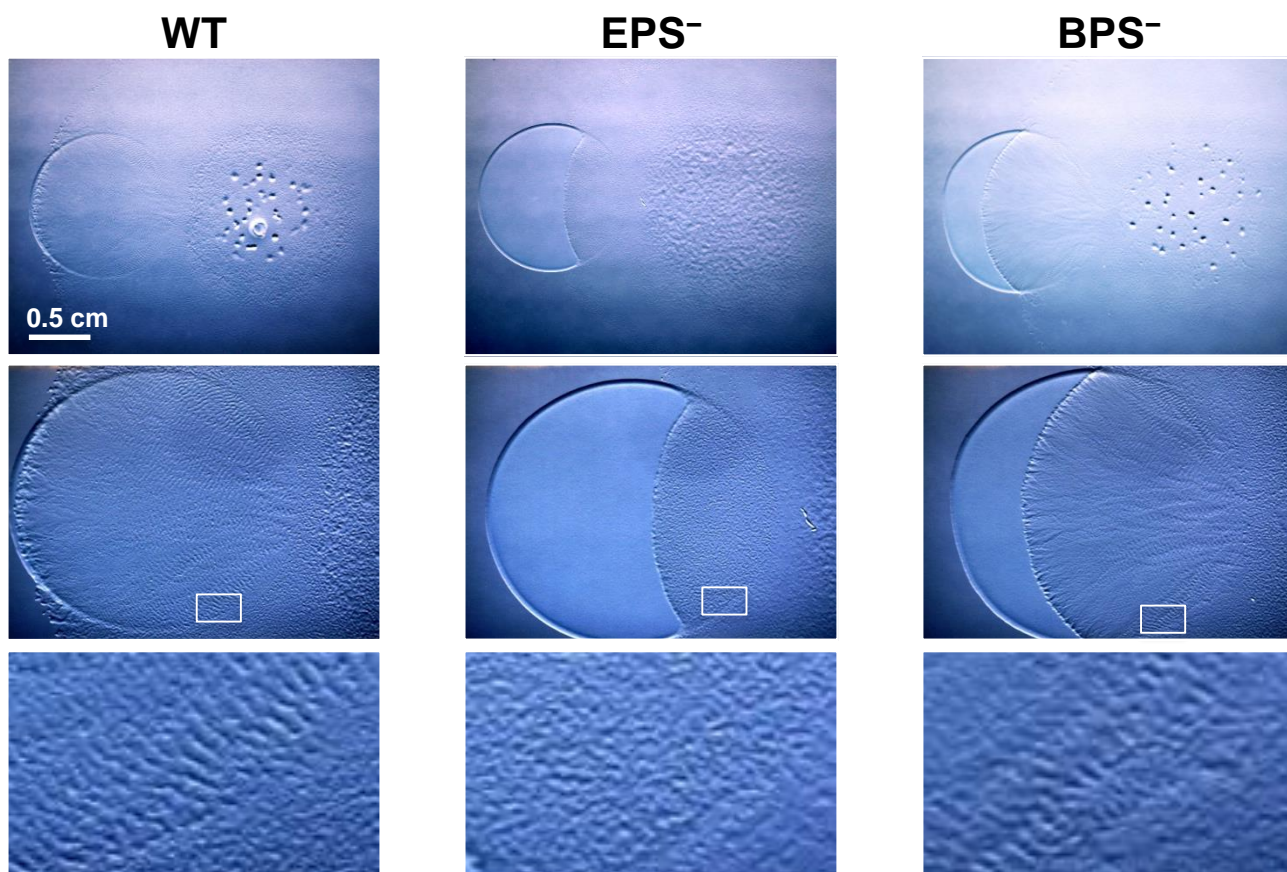

Supplement: S2 Fig — (A) Fruiting body formation phenotypes at different cell densities after growth at 32°C for 72 hours. (B) WT, EPS− (ΔwzaX), and BPS− (ΔwzaB) cells from exponentially growing cultures were resuspended in buffer to a final concentration of OD600 10. Samples were then spotted on developmental media next to an E. coli colony and imaged after 48 hours. The first row contains images of the entire swarm; the second row presents a medium-magnification view of the prey invasion step; the third row presents a high-magnification view of the ripples at sites corresponding to the small boxes on the second row. BPS, biosurfactant polysaccharide; EPS, exopolysaccharide; OD600, optical density at 600 nm; WT, wild type. (PDF) [file pbio.3000728.s002.pdf]

**A****Gel Chromatography**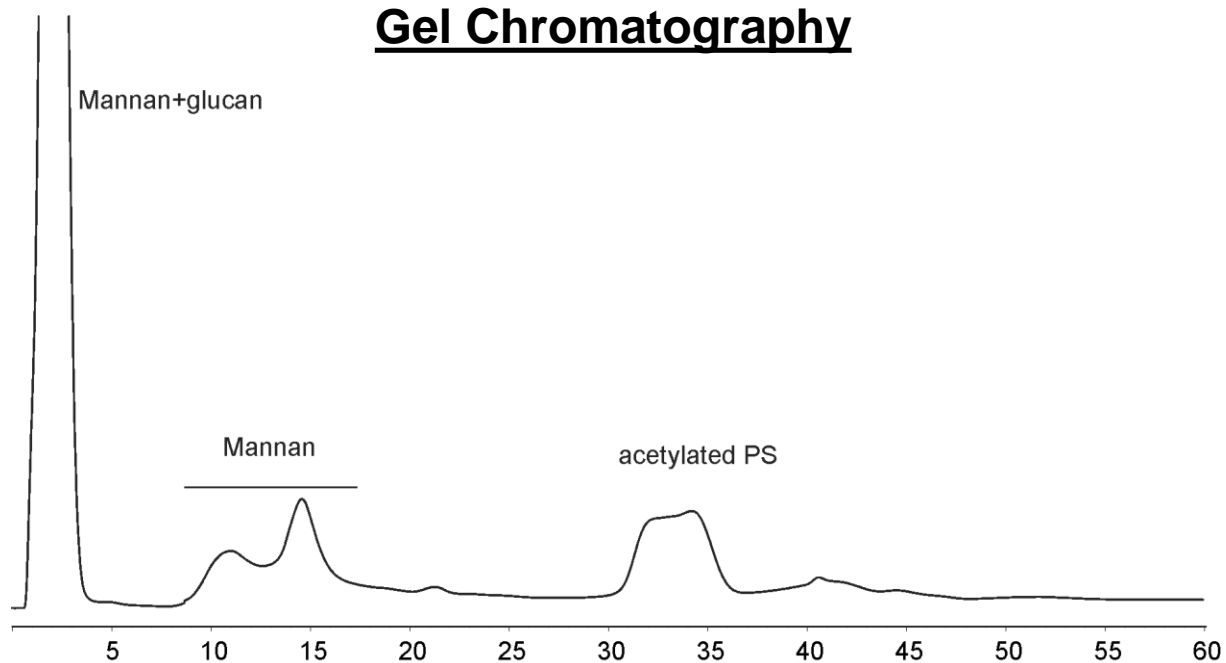**B** **$^1\text{H}$ - $^{13}\text{C}$  HSQC NMR**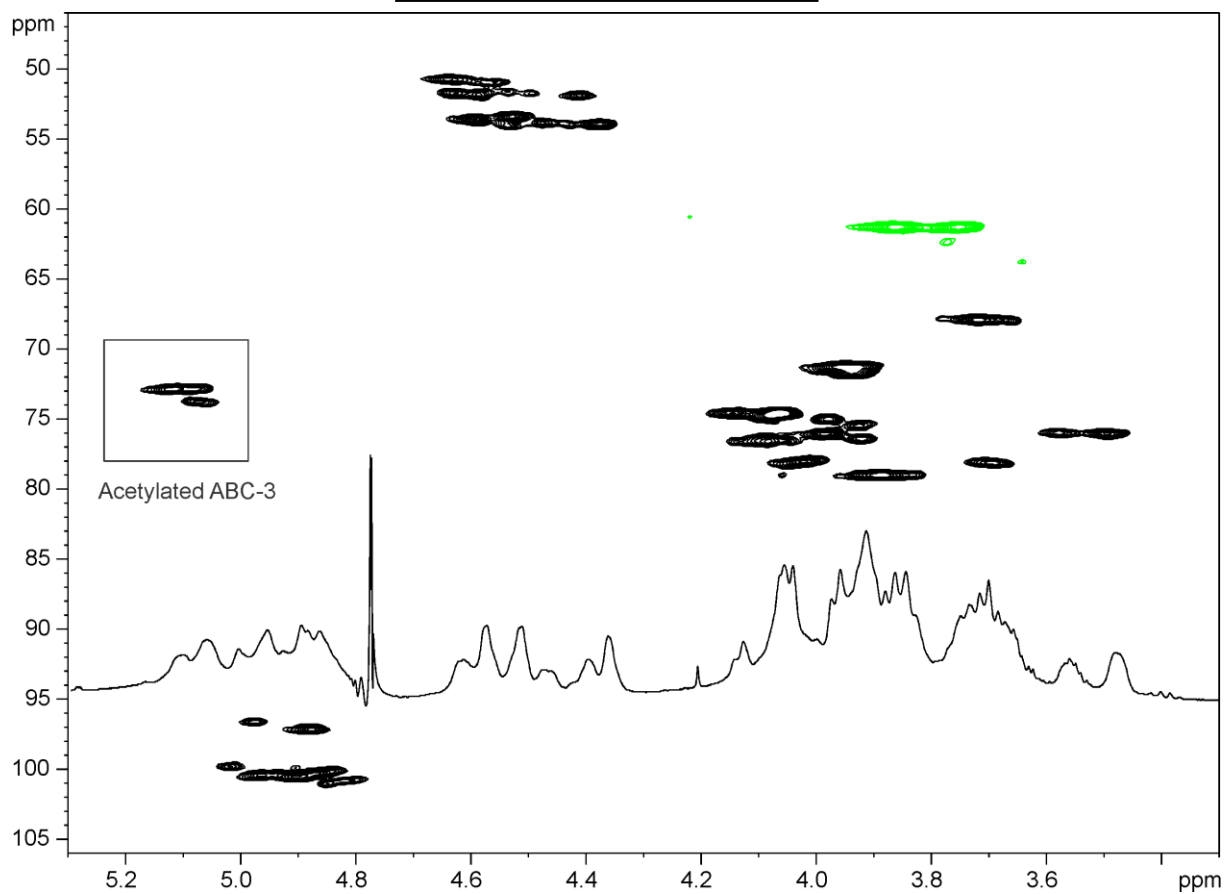

Supplement: S4 Fig — (A) Gel chromatography separation of enriched supernatant from a ΔwzaX ΩpilA culture. (B) 1H–13C HSQC NMR spectrum of acidic PS isolated from ΔwzaX ΩpilA supernatant. Analysis was performed at 25 ºC, 500 MHz. Resonance peak colors: black, C–H; green, C–H2. HSQC, heteronuclear single quantum correlation; PS, polysaccharide. (PDF) [file pbio.3000728.s004.pdf]

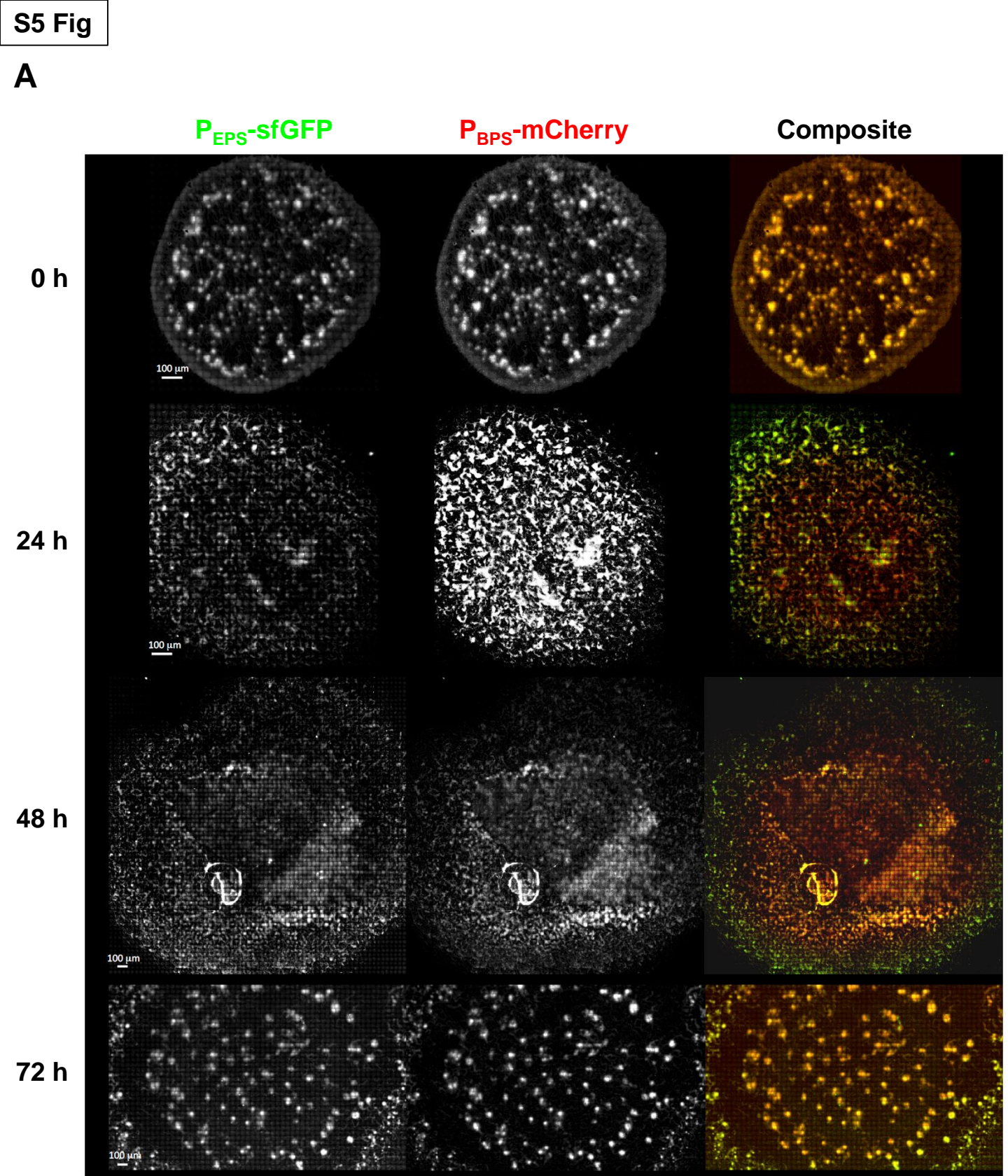

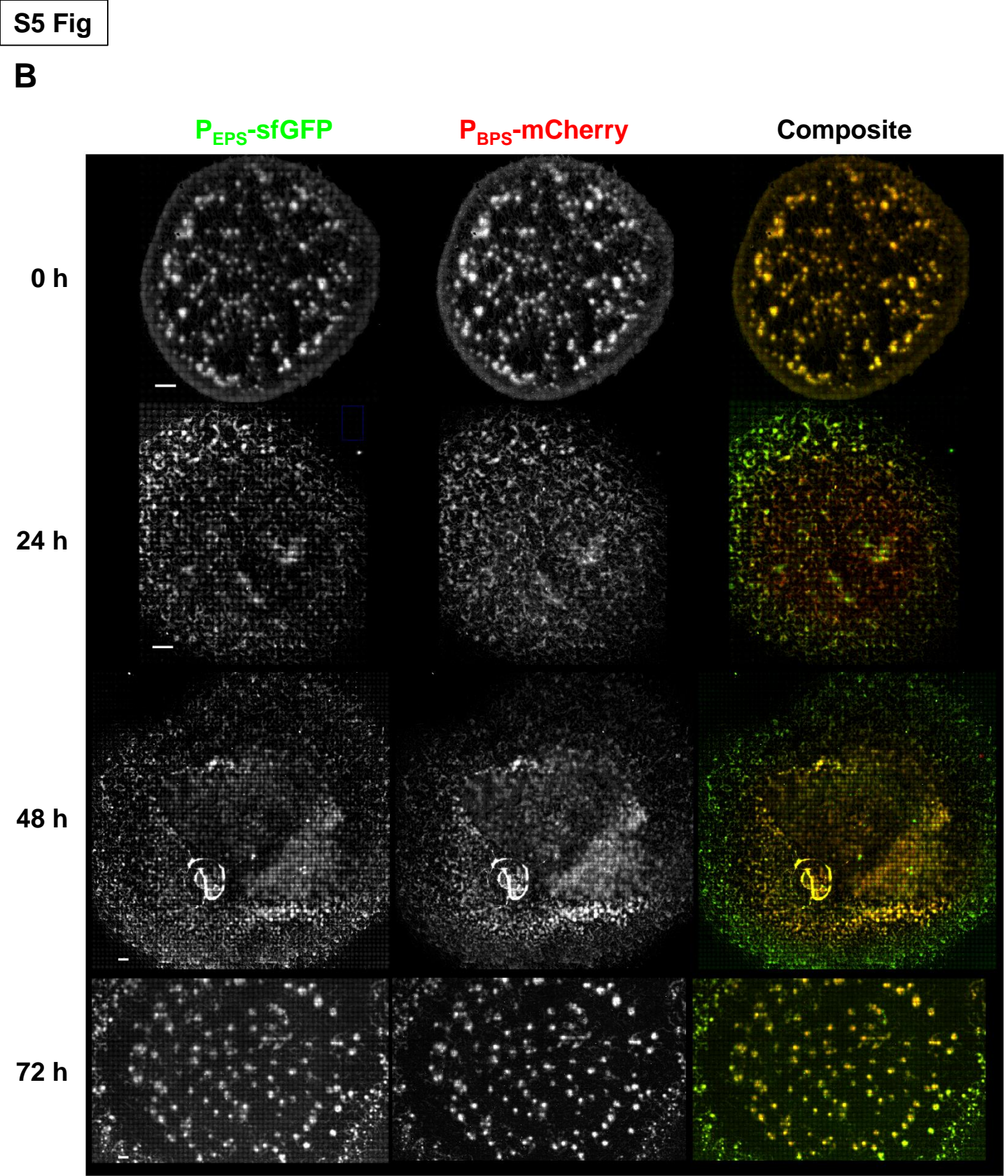

Supplement: S5 Fig — (A) Dual-labeled (PEPS-sfGFP + PBPS-mCherry) WT cells (strain EM709) from exponentially growing cultures were spotted on developmental media at a final concentration of OD600 10.0 and imaged at the indicated time points (scale bar: 100 μm). Images were scaled as described in “Material and methods.” (B) Raw, nonnormalized data displayed in Panel A. BPS, biosurfactant polysaccharide; EPS, exopolysaccharide; OD600, optical density at 600 nm; sfGFP, superfolder green fluorescent protein; WT, wild type. (PDF) [file pbio.3000728.s005.pdf]
