# Supplementary material for: Modulation of bacterial multicellularity via spatio-specific polysaccharide secretion
Source: PLoS Biol. 2020 Jun 9;18(6):e3000728. doi: 10.1371/journal.pbio.3000728 (PMC7310880; doi:10.1371/journal.pbio.3000728)
Supplement: S3 Fig — (A) High-performance anion-exchange chromatography coupled with pulsed amperometric detection for monosaccharide standards versus monosaccharides isolated from submerged-culture supernatants. Strains tested: WT and BPS− (ΔwzaB). (B) Boxplots of trypan blue dye retention to indicate the levels of EPS production in various pilA mutant strains relative to WT. The lower and upper boundaries of the boxes correspond to the 25th and 75th percentiles, respectively. The median (line through center of boxplot) and mean (+) of each dataset are indicated. Lower and upper whiskers represent the 10th and 90th percentiles, respectively; data points above and below the whiskers are drawn as individual points. Asterisks denote datasets displaying statistically significant differences in distributions (p < 0.05) shifted higher (*) than WT, as determined via Wilcoxon signed-rank test performed relative to “100” (i.e., WT); stars denote datasets that are not statistically different relative to “0” (p > 0.05), as determined via Wilcoxon signed-rank test performed relative to “0.” Raw values and detailed statistical analysis are available (S2 Data). (C) Time course of raw surface tension values (via digital-drop tensiometry) from representative submerged-culture supernatants. Strains tested: WT, MASC− (ΔwzaS), BPS− MASC− (ΔwzaB ΔwzaS), EPS− MASC− (ΔwzaX ΔwzaS), EPS− BPS− MASC− (ΔwzaX ΔwzaB ΔwzaS). (D) Representative images of T4P-dependent motility for WT, EPS− (ΔwzaX), BPS− (ΔwzaB), and MASC− (ΔwzaS) strains in the presence/absence of di-rhamnolipid-C14-C14 produced by B. thailandensis E264 (scale bar: 4 mm). Top: Samples spotted (5 μL of OD600 5.0 resuspension in TPM buffer) on a CYE 0.5% agar plate, grown for 72 hours in the absence of rhamnolipid. Bottom: Samples treated analogously to those in top portion of the panel but grown for 72 hours on a CYE 0.5% agar plate pretreated with rhamnolipid. Analysis of these data (Fig 3D) as well as raw values and detailed statistical analysis (S [file pbio.3000728.s003.pdf]

S3 Fig

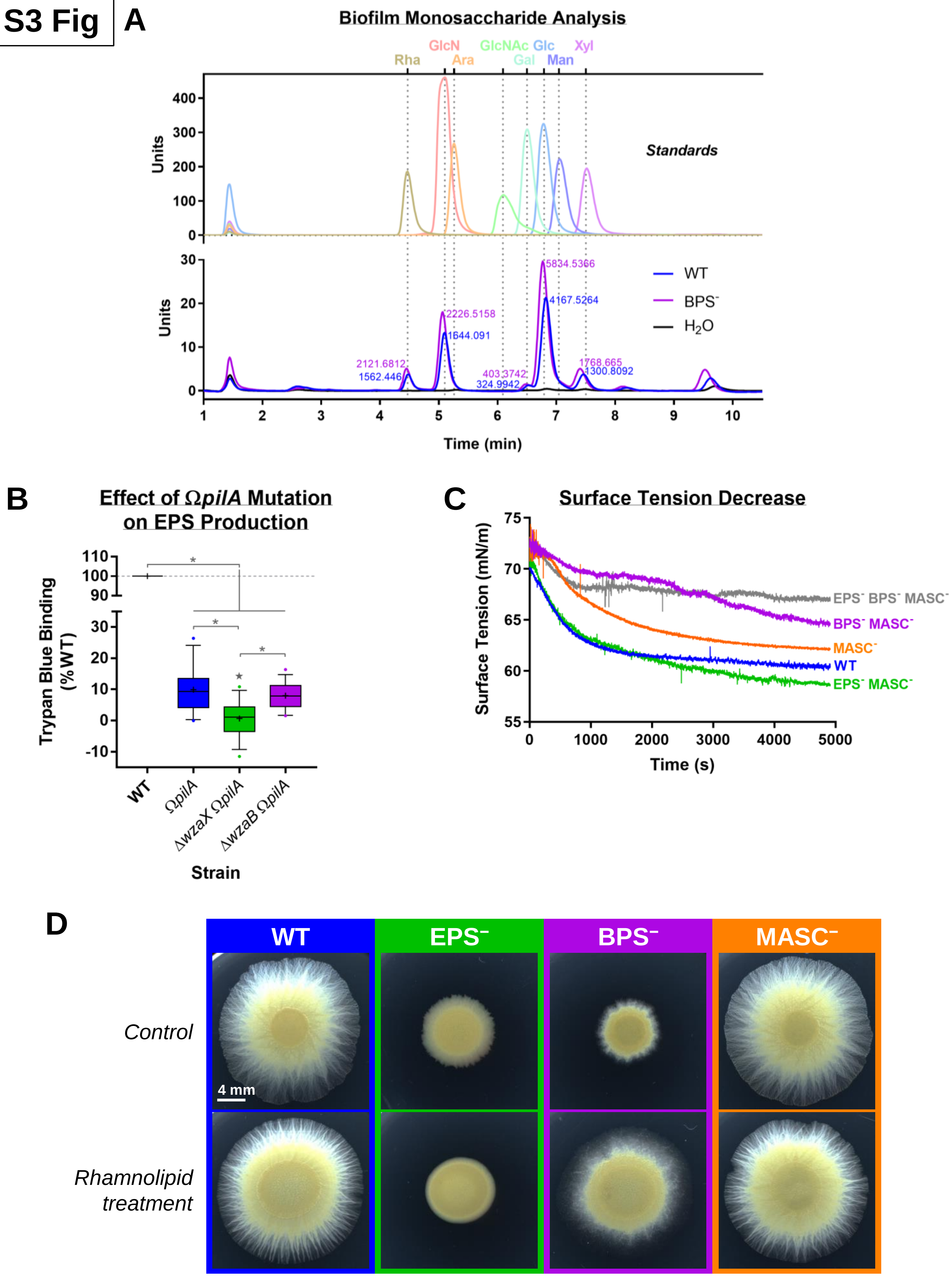

A
Biofilm Monosaccharide Analysis
Rha
GlcN
Ara
GlcNAc
Gal
Glc
Man
Xyl
Standards
Units
400
300
200
100
0
30
20
10
0
WT
BPS-
$H_2O$
5834.5366
4167.5264
2226.5158
1644.091
2121.6812
1562.446
403.3742
324.9942
1768.665
1300.8092
1 2 3 4 5 6 7 8 9 10
Time (min)
B
Effect of ΩpilA Mutation on EPS Production
Trypan Blue Binding (% WT)
110
100
90
30
20
10
0
-10
WT
ΩpilA
ΔwzaX ΩpilA
ΔwzaB ΩpilA
Strain
C
Surface Tension Decrease
Surface Tension (mN/m)
75
70
65
60
55
0 1000 2000 3000 4000 5000
Time (s)
EPS- BPS- MASC-
BPS- MASC-
MASC-
WT
EPS- MASC-
D
WT
EPS–
BPS–
MASC–
Control
Rhamnolipid treatment
4 mm
