# Supplementary material for: Modulation of bacterial multicellularity via spatio-specific polysaccharide secretion
Source: PLoS Biol. 2020 Jun 9;18(6):e3000728. doi: 10.1371/journal.pbio.3000728 (PMC7310880; doi:10.1371/journal.pbio.3000728)
Supplement: S1 Table — BPS, biosurfactant polysaccharide; EPS, exopolysaccharide; MASC, major spore coat. (PDF) [file pbio.3000728.s006.pdf]

S1 Table: Gene nomenclature and annotation analyses								
**Gene Name**	**Former Name [Ref]**	**Locus Tag**	**Protein Length (aa)**	**TMS†**	**Fold Recognition Hit (PDB)***	**Name of PDB Homologue**	**Probability (%)**	**E-value**
wzxX	−	*mxan_7416*	490	14	5T77_A	MOP-family lipid II flippase MurJ	100	1.3E-31
wzyX	*sgnF* [1]	*mxan_7442*	508	12	6BAR_A	Peptidoglycan glycosyltransferase RodA	98.66	3.3E-6
wzcX	*epsV* [1,2]	*mxan_7421*	507	2	4WL1_X	Polysaccharide co-polymerase WzzE	99.9	1.3E-24
wzeX	−	*mxan_7447*	186	−	3CIO_D	Tyrosine-protein kinase Etk, C-terminal Wzc domain	99.41	9.1E-14
wzaX	*epsY* [1]	*mxan_7417*	219	−	2J58_B	Outer-membrane lipoprotein Wza	100.0	1.3E-32
wzxS	*exoM* [3]	*mxan_3260*	506	14	5T77_A	MOP-family lipid II flippase MurJ	100	2.9E-33
wzyS	*exoJ* [3]	*mxan_3026*	426	12	6BAR_A	Peptidoglycan glycosyltransferase RodA	98.75	6.2E-7
wzcS	*exoC* [4]	*mxan_3227*	465	2	4WL1_X	Polysaccharide co-polymerase WzzE	99.93	7.0E-27
wzeS	*btkA* [5] *exoD* [6]	*mxan_3228*	231	−	3LA6_D	Nucleotide-binding domain of Tyr-protein kinase Wzc	99.9	9.3E-24
wzaS	*fdgA* [7] *exoA* [4,6]	*mxan_3225*	190	−	2J58_B	Outer-membrane lipoprotein Wza	100.0	4.4E-32
wzxB	− [4]	*mxan_1035*	506	14	5T77_A	MOP-family lipid II flippase MurJ	100	1.8E-33
wzyB	−	*mxan_1028*	486	12	6BAR_A	Peptidoglycan glycosyltransferase RodA	98.89	2.7E-7
wzcB	*btkB* [8]	*mxan_1025*	710	2	i) aa 2 – 391: 4WL1_X<br>ii) aa 432 – 697: 3CIO_D	i) Polysaccharide co-polymerase WzzE<br>ii) Tyrosine-protein kinase Etk	i) 99.88<br>ii) 99.92	i) 6.2E-24<br>ii) 6.7E-26
wzaB	− [9]	*mxan_1915*	204	−	2J58_B	Outer-membrane lipoprotein Wza	99.95	1.1E-29
†Consensus of OCTOPUS and TMHMM analyses. *Output from HHpred								

[1] Youderian P, Hartzell PL. Transposon insertions of *magellan-4* that impair social gliding motility in *Myxococcus xanthus*. Genetics. 2006. 172(3):1397-1410.
[2] Lu A, Cho K, Black WP, Duan X-Y, Lux R, Yang Z, Kaplan HB, Zusman DR, Shi W. Exopolysaccharide biosynthesis genes required for social motility in *Myxococcus xanthus*. Mol. Microbiol. 2005. 55(1):206-220.
[3] Pérez-Burgos M, García-Romero I, Valvano MA, Søgaard-Andersen L. Identification of the Wzx flippase, Wzy polymerase and sugar-modifying enzymes for spore coat polysaccharide biosynthesis in *Myxococcus xanthus*. Mol. Microbiol. 2020. (advance online). DOI: 10.1111/mmi.14486.
[4] Müller FD, Schink CW, Hoiczyk E, Cserti E, Higgs PI. Spore formation in *Myxococcus xanthus* is tied to cytoskeleton functions and polysaccharide spore coat deposition. Mol. Microbiol. 2012. 83(3):486-505.
[5] Kimura Y, Yamashita S, Mori Y, Kitajima Y, Takegawa K. A *Myxococcus xanthus* bacterial tyrosine kinase, BtkA, is required for the formation of mature spores. J. Bacteriol. 2011. 193(20):5853-5857.
[6] Holkenbrink C, Hoiczyk E, Kahnt J, Higgs PI. Synthesis and assembly of a novel glycan layer in *Myxococcus xanthus* spores. J. Biol. Chem. 2014. 289(46):32364-32378.
[7] Ueki T, Inouye S. Identification of a gene involved in polysaccharide export as a transcription target of FruA, an essential factor for Myxococcus xanthus development. J. Biol. Chem. 2005. 280(37):32279-32284.
[8] Kimura Y, Kato T, Mori Y. Function analysis of a bacterial tyrosine kinase, BtkB, in *Myxococcus xanthus*. FEMS Microbiol. Lett. 2012. 336(1):45-51.
[9] Ducret A, Valignat M-P, Mouhamar F, Mignot T, Theodoly O. Wet-surface-enhanced ellipsometric contrast microscopy identifies slime as a major adhesion factor during bacterial surface motility. Proc. Natl. Acad. Sci. USA. 2012. 109(25):10036-10041.
