# Supplementary material for: Modulation of bacterial multicellularity via spatio-specific polysaccharide secretion
Source: PLoS Biol. 2020 Jun 9;18(6):e3000728. doi: 10.1371/journal.pbio.3000728 (PMC7310880; doi:10.1371/journal.pbio.3000728)
Supplement: S3 Table — (PDF) [file pbio.3000728.s008.pdf]

S3 Table: Bacterial strains and plasmids used in this study				
**Strain Code**	**Strain**	**Genotype**	**Construction**	**Source or Reference**
TM108	*Myxococcus xanthus* DZ2	Wild type	-	Laboratory collection
EM606	Δ*wzxX*	Δ*mxan_7416*	TM108 + pEM440 (pBJ113-Δ*mxan_7416)*	This work
EM616	Δ*wzyX*	Δ*mxan_7442*	TM108 + pEM470 (pBJ113-Δ*mxan_7442)*	This work
EM572	Δ*wzcX*	Δ*mxan_7421/epsV*	TM108 + pEM441 (pBJ113-Δ*mxan_7421)*	This work
EM614	Δ*wzeX*	Δ*mxan_7447*	TM108 + pEM468 (pBJ113-Δ*mxan_7447)*	This work
TM469	Δ*wzaX*	Δ*mxan_7417/epsY*	TM108 + pTM211 (pBJ114-Δ*mxan_7417)*	[1]
TM484	Δ*wzaS*	Δ*mxan_3225/exoA/fdgA*	TM108 + pTM210 (pBJ114-Δ*mxan_3225)*	[1]
EM619	Δ*wzxB*	Δ*mxan_1035*	TM108 + pEM471 (pBJ113-Δ*mxan_1035)*	This work
EM618	Δ*wzyB*	Δ*mxan_1028*	TM108 + pEM472 (pBJ113-Δ*mxan_1028)*	This work
EM588	Δ*wzcB*	Δ*mxan_1025/btkB*	TM108 + pEM462 (pBJ113-Δ*mxan_1025)*	This work
EM615	Δ*wzcB*$_{\mathrm{BYK}}$	Δ*mxan_1025* $_{\text{bp 1392-2091 (BYK domain)}}$	TM108 + pEM469 (pBJ113-Δ*mxan_1025* from aa 465-697)	This work
TM529	Δ*wzaB*	Δ*mxan_1915*	TM108 + pTM212 (pBJ114-Δ*mxan_1915)*	[1]
EM596	Δ*wzcX* Δ*wzaX*	Δ*mxan_7417* Δ*mxan_7421*	TM469 + pEM441	This work
EM592	Δ*wzaX* Δ*wzaB*	Δ*mxan_7417* Δ*mxan_1915*	TM469 + pTM211	This work
EM591	Δ*wzcB* Δ*wzaB*	Δ*mxan_1025* Δ*mxan_1915*	TM529 + pEM462	This work
EM651	Δ*wzaB* Δ*wzaS*	Δ*mxan_1915* Δ*mxan_3225*	TM529 + pTM210	This work
TM488	Δ*wzaX* Δ*wzaS*	Δ*mxan_7417* Δ*mxan_3225*	TM469 + pTM210	This work
TM530	Δ*wzaX* Δ*wzaB* Δ*wzaS*	Δ*mxan_7417* Δ*mxan_1915* Δ*mxan_3225*	TM488 + pTM212 (pBJ114-Δ*mxan_1915)*	This work
TM293	Ω*pilA*	Tetracycline resistance cassette	TM108 + *pilA::tet*, Tcr	Laboratory collection
TM493	Δ*wzaX* Ω*pilA*	Δ*mxan_7417* Ω*pilA*	TM469 + Ω*pilA* chromosomal DNA	This work
TM540	Δ*wzaB* Ω*pilA*	Δ*mxan_1915* Ω*pilA*	TM529 + Ω*pilA* chromosomal DNA	This work
EM693	Δ*wzaX* OMss-sfGFP	Δ*mxan_7417 OMss-sfGFP*	TM469 + pSWU19-*promPilA-OMss-sfGFP*	This work
EM691	Δ*wzaB* IMss-mCherry	Δ*mxan_1915 IMss-mCherry*	TM529 + pSWU19-*promPilA-IMss-mCherry*	This work
EM709	P_{EPS}-sfGFP + P_{BPS}-mCherry	WT P_{EPS}*-sfGFP* + P_{BPS}*-mCherry*	TM108 + pSWU19-*promWzxX-sfGFP* + *promWzcB-mCherry*	This work
EC393	*Escherichia coli* MG1655	F^- *lambda*$^-$ *ilvG*$^-$ *rfb-50 rph-1*	-	Laboratory collection

[1] Ducret A, Valignat M-P, Mouhamar F, Mignot T, Theodoly O. Wet-surface-enhanced ellipsometric contrast microscopy identifies slime as a major adhesion factor during bacterial surface motility. Proc. Natl. Acad. Sci. USA. 2012. 109(25):10036-10041.
