# Supplementary material for: Modulation of bacterial multicellularity via spatio-specific polysaccharide secretion
Source: PLoS Biol. 2020 Jun 9;18(6):e3000728. doi: 10.1371/journal.pbio.3000728 (PMC7310880; doi:10.1371/journal.pbio.3000728)
Supplement: Attachment — Submitted filename: Response to Reviewers.pdf [file pbio.3000728.s012.pdf]

Re: PLOS Biology PBIOLOGY-D-20-00391R1 (Modulation of bacterial multicellularity via spatiotemporal polysaccharide secretion)

Dear Dr. Roberts,

Please find below the various comments, concerns, and suggestions offered by the Editorial Board (*in blue italics*) and our point-by-point response to each (in black).

COMMENTS TO THE AUTHORS:

Dear Dr Islam,

Thank you very much for submitting your manuscript "Modulation of bacterial multicellularity via spatiotemporal polysaccharide secretion" for consideration as a Research Article by PLOS Biology. As with all papers reviewed by the journal, yours was evaluated by the PLOS Biology editors as well as by an Academic Editor with relevant expertise and in this case by three independent reviewers.

Based on the reviews, we will probably accept this manuscript for publication, assuming that you will modify the manuscript to address the remaining points raised by the reviewers.

Response: Given the unprecedented global situation, we are particularly grateful to the journal staff and peer reviewers for the efforts in processing our submission, as well as the overall positive reception of our manuscript. We have addressed the various comments (outlined below) throughout the manuscript to facilitate its acceptance for publication.

IMPORTANT:

a) The Academic Editor asked me to emphasise the following "Some of the claims of first are apparently exaggerated (two reviewers point this out). This certainly needs to be toned down or clarified."

Response: (i) We have heeded this advice and toned down certain claims of first, as well as provided additional explanations where warranted. More details can be found in the Responses to the specific Reviewer comments below. (ii) So as not to "over"-claim, we have also tweaked the title and text of the manuscript to emphasize the *spatial* differences in EPS and BPS expression profiles (i.e. the former at the periphery and the latter in the swarm interior); these were doubly-verified via microscopy and flow cytometry for the standard 48 h time point, with the same relative profile seen across different microscopy time points (Fig 6 and S5 Fig). We do not however have quantitative transcriptional profiling data that show absolute fluctuations in EPS- and BPS-pathway levels across sequential time points that would allow us to safely conclude that there is specifically *temporal* regulation (in addition to the spatial regulation we have clearly detected). Moreover, the lone publication in which RNAseq has been used to transcriptionally profile the complete *M. xanthus* developmental cycle (Muñoz-Dorado *et al.* 2019. *eLife* 8:50374) yields incomplete results with respect to EPS- and BPS-pathway expression levels over time. Thus as mentioned, we have reduced discussion of temporal changes to err on the side of caution. This has in no way though detracted from the novelty, thoroughness, and

excitement for our story, as seen in the highly-positive reaction to our manuscript by all three peer Reviewers.

b) Please also make sure to address the Data Policy requests noted further down the email.

Response: The various requests have been fulfilled. Please refer to the details below.

We expect to receive your revised manuscript within two weeks. Your revisions should address the specific points made by each reviewer. In addition to the remaining revisions and before we will be able to formally accept your manuscript and consider it "in press", we also need to ensure that your article conforms to our guidelines. A member of our team will be in touch shortly with a set of requests. As we can't proceed until these requirements are met, your swift response will help prevent delays to publication.

Copyediting
Upon acceptance of your article, your final files will be copyedited and typeset into the final PDF. While you will have an opportunity to review these files as proofs, PLOS will only permit corrections to spelling or significant scientific errors. Therefore, please take this final revision time to assess and make any remaining major changes to your manuscript.

NOTE: If Supporting Information files are included with your article, note that these are not copyedited and will be published as they are submitted. Please ensure that these files are legible and of high quality (at least 300 dpi) in an easily accessible file format. For this reason, please be aware that any references listed in an SI file will not be indexed. For more information, see our Supporting Information guidelines:
https://journals.plos.org/plosbiology/s/supporting-information

Published Peer Review History
Please note that you may have the opportunity to make the peer review history publicly available. The record will include editor decision letters (with reviews) and your responses to reviewer comments. If eligible, we will contact you to opt in or out. Please see here for more details:
https://blogs.plos.org/plos/2019/05/plos-journals-now-open-for-published-peer-review/

Early Version
Please note that an uncorrected proof of your manuscript will be published online ahead of the final version, unless you opted out when submitting your manuscript. If, for any reason, you do not want an earlier version of your manuscript published online, uncheck the box. Should you, your institution's press office or the journal office choose to press release your paper, you will automatically be opted out of early publication. We ask that you notify us as soon as possible if you or your institution is planning to press release the article.

Protocols deposition
To enhance the reproducibility of your results, we recommend that if applicable you deposit your laboratory protocols in protocols.io, where a protocol can be assigned its own identifier (DOI) such that it can be cited independently in the future. For instructions

see: https://journals.plos.org/plosbiology/s/submission-guidelines#loc-materials-and-methods

**Submitting Your Revision*
To submit your revision, please go to https://www.editorialmanager.com/pbiology/ and log in as an Author. Click the link labelled 'Submissions Needing Revision' to find your submission record. Your revised submission must include a cover letter, a Response to Reviewers file that provides a detailed response to the reviewers' comments (if applicable), and a track-changes file indicating any changes that you have made to the manuscript.*

Please do not hesitate to contact me should you have any questions.

*Sincerely,
Roli Roberts*

*Roland G Roberts, PhD,
Senior Editor, PLOS Biology*
--
DATA POLICY:

You may be aware of the PLOS Data Policy, which requires that all data be made available without restriction: http://journals.plos.org/plosbiology/s/data-availability. For more information, please also see this editorial: http://dx.doi.org/10.1371/journal.pbio.1001797

Note that we do not require all raw data. Rather, we ask that all individual quantitative observations that underlie the data summarized in the figures and results of your paper be made available in one of the following forms:

1) Supplementary files (e.g., excel). Please ensure that all data files are uploaded as 'Supporting Information' and are invariably referred to (in the manuscript, figure legends, and the Description field when uploading your files) using the following format verbatim: S1 Data, S2 Data, etc. Multiple panels of a single or even several figures can be included as multiple sheets in one excel file that is saved using exactly the following convention: S1_Data.xlsx (using an underscore).

2) Deposition in a publicly available repository. Please also provide the accession code or a reviewer link so that we may view your data before publication.

Regardless of the method selected, please ensure that you provide the individual numerical values that underlie the summary data displayed in the following figure panels as they are essential for readers to assess your analysis and to reproduce it: Figs 2A, 3ABCDE, 5B, 6B, and S3B. I note that data for 2A, 3D, 5B are available in Supp Table 4, and that data for 3A and S3B are in Supp Table 5. These two files should be renamed Supp Data 1 and Supp Data 2 and cited in the relevant Fig legends. I'm not seeing data for Figs 3BCE or 6B; please supply these or clrify where they can be found. NOTE: the numerical data provided should include all replicates AND the way in which the plotted mean and errors were derived (it should not present only the mean/average values).

Response: We have now provided three Supplementary Data tables, in Excel format:

- **S1_Data.xlsx →** Source data and statistical analyses for Figs 2A, 3D, 5B (all datasets involving swarm surface area quantitation)
- **S2_Data.xlsx →** Source data and statistical analyses for Figs 3A, 3B, 3E, S3B (all datasets involving spectrophotometer readings)
- **S3_Data.xlsx →** Source data and statistical analyses for Figs 3C, 6B, and S3C (all datasets involving surface tension and flow cytometry quantitation)

Regarding the functional complementation *in trans* of our BPS^{-} mutant with exogenous rhamnolipid biosurfactant, we have also provided a representative set of images (S3D Fig) for the quantification data that was already presented in the main text (Fig 3D) (and for which raw values are located in S1_Data).

Please also ensure that figure legends in your manuscript include information on where the underlying data can be found, and ensure your supplemental data file/s has a legend.

Response: We have modified the relevant main-text figure legends accordingly. We have also added a separate Supporting Information legends section to the manuscript file.

Please ensure that your Data Statement in the submission system accurately describes where your data can be found.

Response: All data are either included in the main-text manuscript or as part of the Supporting Information.

REVIEWERS' COMMENTS:

Reviewer #1:

The manuscript entitled "Modulation of bacterial multicellularity via spatiotemporal polysaccharide secretion" by Islam et al. describes the genetic determinants of three major polysaccharides secreted by the social bacterium M. xanthus. These three polysaccharides are produced by three different Wzx/Wzy-dependent pathways and expressed differentially through time and space. One of these polysaccharides, BPS, has not been previously described. Its genetic basis and its chemical composition are determined in detail, as wells as the potential implications for sociality in M. xanthus.

This manuscript is very complete in the characterization of a novel polysaccharide. The work is beautifully performed, the paper is very easy to read, clearly written and the figures are exhaustive and illustrate the results precisely. There is a nice balance between genetics, chemistry and social phenotypes. The authors should be commended for this.

Response: We are very grateful for the Reviewer's enthusiasm and praise regarding this submission.

I strongly believe that this study is of great quality and, despite the fact that in its current state it may be of interest to a narrow readership (microbiologists working with M. xanthus) it should be accepted for publication with minor changes (see comments below).

Comments

1. I would tone down the claims (abstract and discussion) that this is the first report of an EPS synthesized by a Wzx/Wzy pathway with biosurfactant properties. There have been some capsular polysaccharides (synthesized by the Wzx/Wzy pathway) described to inhibit the formation biofilm (see review https://doi.org/10.1111/j.1462-2920.2012.02810.x) due to their surface-active properties.

Response: We agree with the Reviewer's comment and have removed the statement that this is the first finding of a Wzx/Wzy-dependent pathway product with biosurfactant properties (please also refer to comment #1 of Reviewer #2). In addition, we have added a very short Discussion paragraph explicitly describing the current inadequacies in the broader scientific literature concerning use of the terms "bioemulsifier" and "biosurfactant", as the two describe distinct properties, but are typically incorrectly used interchangeably, leading to much ambiguity and confusion in reported findings (*see Lines 529-538 in the revised version*).

2. I found extremely interesting the fact that the genetic basis for the three polysaccharides is broken up and dispersed in the M. xanthus genome, specially, when in most genomes, Wzx/Wzy-dependent EPS genes are grouped in a contiguous gene cluster, as elegantly shown in Figure 1B. I believe the manuscript would gain relevance for evolutionary biologists if this were a little bit more discussed. Do the authors have any theory? Are there insertions found breaking these gene clusters flanked by mobile genetic elements like transposases, prophages, etc..? Is the GC content of these genes more AT-rich than the rest of the genomes, suggesting that they have been acquired by horizontal-gene transfer?

Response: We agree with the Reviewer that the genome complexity of the polysaccharide synthesis clusters studied herein are of considerable interest. We continue to study Myxobacterial origins with regard to their genome expansion/procurement, either from gene duplication or horizontal gene transfer; both of these phenomena will be helpful in understanding how myxobacteria developed their various physiological properties as compared to their delta-proteobacterial counterparts. Another future avenue of investigation would be to understand from where these polysaccharide synthesis clusters originated during evolution. Cursory analyses of genes nearby the synthesis clusters reveal slight variations in GC content, but no correlation or any large stretch of genes with low GC-content have been detected (*M. xanthus* genome is intrinsically GC-rich). There are also a few transposable elements detected nearby, but Myxobacterial genomes are littered with such elements, so dedicated phylogenetic studies and papers (such as Holt *et al.* 2020. *ISME J*, advance online, [doi: 10.1038/s41396-020-0628-0]) will need to be devoted to answering these questions.

3. I believe that some measure of similarity between the identified proteins in M. xanthus and other genome(s) should be reported, especially since the cut-off used by blastp is, in my opinion, fairly low (35% query coverage and 35% similarity). I would not call this "stringent" (L510).

Response: We share the Reviewer's concerns regarding the lower-than-normal cut-offs used, but this was by necessity and design. Unfortunately, proteins in Wzx/Wzy-dependent assembly pathways display notoriously low sequence identity/similarity to other homologues, even between strains of the same species (Islam and Lam. 2014. *Can J Microbiol* 60:697-716). The low sequence identity between (e.g.) the *wzx*/*wzy* genes has even been exploited for the development of numerous molecular serotyping schemes. It is for this very reason that the BLASTp-identified hits were subsequently supported by a) α-helical transmembrane segment (TMS) predictions, and b) fold-recognition analyses against existing structures in the Protein Data Bank, as originally supplied in S2 Table. Each assembly protein has a characteristic TMS profile, as well as one or more structurally-characterized related proteins with an analogous fold. To condense the exposition in this Response, we have expanded the relevant section of the Materials and Methods section to explain our use of the lower cut-off values (*see Lines 655-658 in revised version).*

4. Concerning the complementation in swarming between BPS- and EPS-, I am a little bit confused with the authors conclusion that BPS is not a shared good, when (i) it is secreted to the environment, and (ii) 10% of BPS+ cells in a mix can complement swarming deficiency of the group. Further, the authors show different combinations of 90:10 BPS-:EPS- and viceversa, but do not comment on the results. It may not be clearer to the reader why they performed this experiment and how to interpret its results. In their opinion, why does 90:10 BPS-:EPS- fully complement swarming, but not 10:90 BPS-:EPS-? This has to be discussed further.

Response: We understand the Reviewer's concern. When we first observed the results of the mixing experiments, we were excited by the idea that both the EPS^{-} and BPS^{-} strains could trans-complement each other. However, by fluorescently labelling the two strains in the mixing experiment, we found that too few BPS^{-} cells are present at the colony edge to conclude that BPS^{-} cells are functionally complemented by BPS production by EPS^{-} cells. These direct observations down to the single-cell level led us conclude that BPS^{-} cells are very poorly complemented and that only EPS is a shared good. To promote clarity, we have modified Fig 5A and 5B to show 100% BPS^{-}, 50% BPS^{-}:50% EPS^{-}, 100% EPS^{-}, and WT (i.e. removed the 9:1 and 1:9 datasets); in this way, the fluorescence data in Fig 5C can be more easily interpreted within the context of the preceding Fig 5A and 5B panels.

Reviewer #2:

This manuscript describes the genetic organization of genes involved in the synthesis and transport of three different polysaccharides produced by Myxococcus xanthus through distinct Wzx/Wzy pathways. Islam, Alvarez, Saidi et al then focused their works on the characterization of one of these pathways, the BSP pathway, which is required for proper swarming. They analyzed its chemical structure and demonstrated that BSP is a secreted polysaccharide with

biosurfactant properties. The authors also showed that single mutants defective in BPS or the cell surface polysaccharide EPS can complement each other in trans when present in a mixed population. Furthermore, they showed that these mutants organize differently in space within these communities and that expression of promoters for EPS and BSP genes are also spatially and temporarily distinct.

The work presented here is solid and well done, and the main conclusions are justified. Overall, the manuscript is also well written. Although I did not find major flaws with the work, I believe that this manuscript would be better suited for a specialized journal, as, in my opinion, it does not meet the description of PLOS Biology publications being of exceptional significance, originality, and relevance.

Response: As with the comments from Reviewer #1, we are very appreciative of Reviewer 2's positive response to this manuscript.

Below are some minor points I suggest the authors address to improve their manuscript:
1. The authors highlight (in the Abstract and the Discussion) the fact that the biosurfactant polysaccharide described here (BSP) is produced by a Wzx/Wzy pathway, but they failed to convey why a reader should find a link between a Wzx/Wzy pathway and a surfactant polysaccharide so novel and interesting. Moreover, I am confused about the fact that they state that this link is novel because, in the Discussion, they refer to a biosurfactant polysaccharide that Acinetobacter makes via a Wzx/Wzy pathway. Please clarify if BSP is indeed the first described biosurfactant that is produced by a Wzx/Wzy pathway and why readers should care about this fact.

Response: We appreciate the Reviewer's comment and have removed the statement that the finding of a Wzx/Wzy-dependent pathway product with biosurfactant properties is novel (please also refer to comment #1 of Reviewer #1, as well as our response).

2. Lines 200-204: The authors state: "The only mutants that showed slightly divergent motility and developmental phenotypes compared to other respective EPS- and BPS-pathway mutants were ΔwzxX and ΔwzxB (Fig 2); this is consistent with wzx mutations in one pathway having the potential to affect the biosynthesis of polysaccharides from unrelated pathways (also requiring UndPP-linked precursors) due to depletion of available UndP." To what differences in phenotypes are they referring? The difference in Fig. 2B? None of the other panels show anything of significance, at least to the non-Myxo experts like me. In fact, I think that the authors should consider removing their statement because they do not report the same for the ΔwzyB mutant, which should behave similarly to the ΔwzxB mutant since it would also lead to UndP depletion. I therefore suggest the authors remove these sentences, unless they clearly describe differences that are significantly distinguishing the ΔwzxX and ΔwzxB from the rest of mutants. In contrast, I agree with their argument to explain the differences for wzxB and wzyB mutants in Fig 3A (lines 219-225).

Response: We agree with the Reviewer's suggestions and have removed the statement in question with respect to the motility and developmental phenotypes (Fig 2A and 2B). In

addition, we appreciate that the Reviewer agrees with our rationale for the lowered Trypan Blue binding by the *wzxB* and *wzyB* mutants (Fig 3A).

3. Lines 228-231: The authors conclude that "the effect of BPS may be downstream to that of EPS" because the dye-binding profile of the EPS- and BPS-pathway double mutant ΔwzaX ΔwzaB matched that of the EPS pathway ΔwzaX single-mutant. These genetic results indicate that these are independent pathways. They do not imply order.

Response: We agree with the Reviewer and have modified the statement to read "*These genetic results reinforce the notion that the EPS and BPS biosynthesis pathways are independent of each other*" *(see Lines 272-273 in revised version).*

4. Fig. 3C: Why are the two slopes of the EPS- MASC- double mutant so different? Is that mutant stable or the assay so variable?

Response: Given the nature of the samples (overlay liquid from surface-grown swarms), there is inherent variability between experiments performed on different days. However, comparison of mutants grown and examined at the same time reveals a clear trend, thus the comparisons *within* the same experiment are highly informative. After eliminating any potential confounding effects from MASC production, any MASC^{-} strain (with or without the capacity for EPS production) was still able to reduce surface tension, except for when a BPS^{-} mutation was introduced. In these instances, different mutants in which BPS^{-} mutations were added were no longer able to reduce surface tension (Fig 3C).

5. Lines 386-387: The authors state that BPS is a T4P-regulated polysaccharide. There is no evidence that the effect of T4P on emulsion clearance (Fig. 3E) is occurring through a regulatory mechanism. They should rephrase their statement.

Response: We agree that use of the term "regulation" is not directly supported at this stage by the data presented and have toned-down our phrasing. The link between the presence of a functional T4P and the regulation of EPS production in *M. xanthus* has long been established (Black *et al.* 2006. *Mol Microbiol* 61:447-456), and our data herein indicate that inactivation of the T4P results in high levels of BPS production (Fig 3E). We have thus amended the text in the Abstract *(see Lines 39-40 in revised version)*, Introduction *(see Lines 151-152 in revised version)*, Results *(see Lines 333 and 383 in revised version)*, and Discussion *(see Lines 516-517 in revised version)* to mention "inhibition" instead of "regulation".

6. Fig. S2B: If EPS mutants are severely defective in swarming motility, what drives their invasion into the E. coli colony? The residual swarming T4P motility or some other type of motility?

Response: As the T4P-dependent motility system and the Agl–Glt gliding motility system are genetically distinct (and not inter-dependent), cells can still glide on hard surfaces used for predation assays in the absence of T4P-dependent motility and EPS. All other tests are

performed on soft agar where only T4P-dependent motility requiring EPS is active. It is also worth noting that the lack of EPS production does not completely shut down T4P-dependent motility. Instead, T4P-dependent motility just becomes *very* inefficient. As per Fig 2A, cells unable to produce a T4P (i.e. *pilA* mutant) are unable to swarm outward, whereas all EPS-pathway mutants (i.e. *wzxX*, *wzyX*, *wzcX*, *wzeX*, *wzaX* mutants) could still swarm outward, but at a greatly reduced capacity.

Reviewer #3:
[identifies himself as Ákos T. Kovács]

It is a very well written manuscript that provides an insight into polysaccharide production in Myxococccus xanthus, identifying a biosurfactant polysaccharide in addition to the already known other two apparatuses involved in polysaccharide production. Analyzing mutants reveals that disruption of genes related to BPS production alters spreading motility/swarming, displays aggregative phenotype, and indirect assays suggest that BPS is not cell surface associated unlike EPS. The structure of BPS is further revealed, and the spatial production of EPS and BSP are described in swarming colonies. This is a great contribution to the field of bacterial surface motility, even outside the Myxococcus area.

Response: We are very pleased with the Reviewer's reaction to our story and appreciate his acknowledgement of the significance of this work to the broad field of bacterial surface motility.

The conclusions from the experiments described in L337-L353 (i.e. EPS is public, while BPS private good) are not consistent with the previous observations described in the manuscript, namely, EPS seems to be cell bound, while BPS is not. This can be simply tested by supplying the BPS- strain with BPS overproducing supernatant (DwzaB OpilA strain). In addition, the lack of BPS- strain surface expansion could be also due to hyper-aggregating phenotype, described earlier. Thus, the conclusions from these experiments should be smoothen, and alternative hypothesis also mentioned.

Response: We understand the Reviewer's concerns.

(i) When we first observed the results of the mixing experiments, we were excited by the idea that both the EPS$^-$ and BPS$^-$ strains could trans-complement each other. However, by fluorescently labelling the two strains in the mixing experiment, we found that too few BPS$^-$ cells are present at the colony edge to conclude that BPS$^-$ cells are functionally complemented by BPS production by EPS$^-$ cells. These direct observations down to the single-cell level led us conclude that BPS$^-$ cells are very poorly complemented and that only EPS is a shared good. To promote clarity, we have modified Figure 5A+B to show 100% BPS$^-$, 50% BPS$^-$:50% EPS$^-$, 100% EPS$^-$, and WT (i.e. removed the 9:1 and 1:9 datasets); in this way, the fluorescence data in Fig 5C can be more easily interpreted within the context of the preceding "A" and "B" panels.

(ii) With regards to additional experiments (given the global situation, and considering that the two main labs responsible for this paper are located in Canada and France, both countries under extended lockdown procedures), we are unable for the foreseeable future to add

further wet lab data to the current manuscript package, which as commented on by other Reviewers is quite comprehensive.

(iii) We agree that a contributing factor to the lack of swarm spreading in a BPS^{-} mutant (Fig 2A and 2B) may be due to the hyper-aggregative phenotypes observed (S2A Fig). The manuscript has thus been modified to raise this possibility *(see Lines 237-238 in revised version).*

Minor:

L174: at this point of the manuscript, the functionality of the third apparatus is not proven, thus correct: "clusters all encode respective Wzx, Wzy, Wzc, and Wza protein homologues"

Response: We agree with the Reviewer's statement and have amended the text to read *"Thus, the EPS, MASC, and BPS clusters all encode putative Wzx, Wzy, Wzc, and Wza proteins…" (see Lines 205-206 in revised version).*

L374: I assume the authors used flow cytometer technique to detect fluorescence (as stated correctly in the methods) and did not actually sort the cells (independently using an instrument with sorting ability)

Response: The Reviewer is correct and we thank him for pointing out this misstatement. All mentions of this dataset in the text have been modified to read "flow cytometry" *(see Lines 504, 507, 818, and 820 in revised version).*
